# Advancing Sarcopenia Management Through Integrated Clinical-Research Translation and Innovation

**DOI:** 10.1093/geroni/igaf122.1282

**Published:** 2025-12-31

**Authors:** Yaomin Hu

**Affiliations:** Renji Hospital, Shanghai, Shanghai, China (People’s Republic)

## Abstract

Sarcopenia is closely linked to aging, driving our comprehensive clinical and scientific efforts to address this condition through a multidimensional integration of clinical translation and technological innovation. We established a database and biobank for sarcopenia, which formed the foundation for leading the development of the 2024 Chinese Expert Consensus on the Construction of Standardized Biobank and Database for Sarcopenia. This initiative has fostered a collaborative medical consortium network for sarcopenia management. Our self-developed AI diagnostic system, Muscle and Fat Intelligent Analysis System (MFIAS), automates CT-based muscle quantification, significantly improving analytical efficiency compared to the traditional manual approach, while shifting from single-parameter to multidimensional metrics analysis. The newly constructed skeletal muscle rehabilitation unit employs isokinetic dynamometry testing and training systems, demonstrating short-term improvements in physical performance scores and metabolic parameters through personalized treatment protocols. In addition, in collaboration with the School of Basic Medical Sciences, we have established a basic science research team to investigate the molecular mechanisms underlying skeletal muscle aging. These innovations align with global trends in precision geriatrics, offering scalable solutions for promoting healthy aging for the ever-growing older adult population. By redefining sarcopenia as a modifiable aging trajectory rather than an inevitable decline, our work provides an actionable framework for early intervention and multidisciplinary care, addressing one of the most pressing challenges in gerontological health.

